# Comparing the effects of two initial specimen diversion techniques on blood culture contamination rates and clinical outcomes—a multicenter study

**DOI:** 10.1017/ash.2024.391

**Published:** 2024-11-11

**Authors:** Anthony Febres-Aldana, Patrycja Ashley, Eva Amenta, Megan Duffey, Theresa Sepulveda, Corina Lopez, Sabra Shay, Miriam Barrett, Todd Lasco, Takei Pipkins, Margaret Reed, Bradley Lembcke, Mayar Al Mohajer

**Affiliations:** 1 Department of Medicine, Section of Infectious Diseases, Baylor College of Medicine, Houston, TX, USA; 2 Division of Infectious Disease, Moffitt Cancer Center, Tampa, FL, USA; 3 Department of Medicine, Baylor College of Medicine, Houston, TX, USA; 4 Clinical Intelligence, TheraDoc Analytics, Premier Inc., Charlotte, NC, USA; 5 Baylor St. Luke’s Medical Center, Houston, TX, USA; 6 Department of Pathology, Baylor College of Medicine, Houston, TX, USA

## Abstract

Although the initial specimen diversion device (ISDD) has been shown to reduce blood culture contamination (BCC) rates, its impact on clinical outcomes remains unclear. This multicenter study showed that ISDD significantly decreased BCC. However, there was no reduction in length of stay, days of therapy, or central line-associated bloodstream infections.

## Introduction

Blood cultures (BCx) are the gold standard for diagnosing bacteremia and fungemia; however, blood culture contamination (BCC) due to suboptimal techniques can compromise their diagnostic utility.^1^ BCC can lead to unnecessary follow-up testing and excessive exposure to antibiotics; in addition, it has been associated with false-positive central line-associated bloodstream infections (CLABSI), extended hospital stays of 1–8.4 days, and a projected economic impact of $3,073–$4,818 per instance.^1,2^

The Initial Specimen Diversion Technique (ISDT), which involves discarding a small amount of blood before inoculating the remaining blood into culture bottles, was developed to reduce BCC beyond traditional techniques. Three versions of ISDT exist, including an open technique and two commercially available initial specimen diversion devices (ISDD).^1^ Implementing ISDD has led to a substantial reduction in BCC and a projected decrease in hospital length of stay (LOS) and costs compared with standard care (without ISDD).^1,3^ Our study aimed to evaluate the impact of implementing one ISDD on BCC and clinical outcomes compared to the open technique.

## Methods

**Figure A1.**
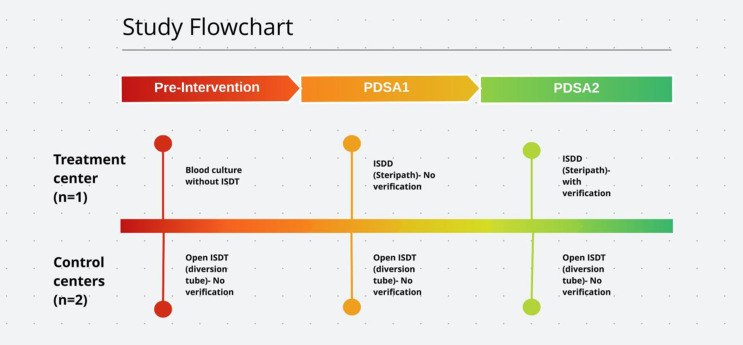
**Study Flow Chart.** This chart shows the different phases of the study across the treatment and control arms. PSDA: Plan-Do-Study-Act; ISDT: Initial Specimen Diversion Technique; ISDD: Initial Specimen Diversion Device.

This non-randomized prospective controlled study was conducted across three hospitals (Figure A1). Center 1, an academic center, was assigned as the treatment arm. During the pre-intervention period (08/2020–05/2021), BCx were collected without ISDT. In the first Plan-Do-Study-Act (PDSA1) cycle (05/2021–01/2022), we implemented an ISDD (Steripath); however, the adoption initially had a low uptake (estimated <25%) due to the absence of process metrics. Therefore, a second PDSA (PDSA2) was implemented (01/2022–08/2022), requiring the submission of the ISDD wrapping label alongside the BCx sample for processing. Centers 2 and 3 (community hospitals) represented the control arm utilizing open ISDT (diversion tube) throughout the study (started in 2019). They did not have a process measure to verify ISDT use. In all three centers, BCx were collected by trained nurses using the same antiseptic procedures.

**Figure 1.**
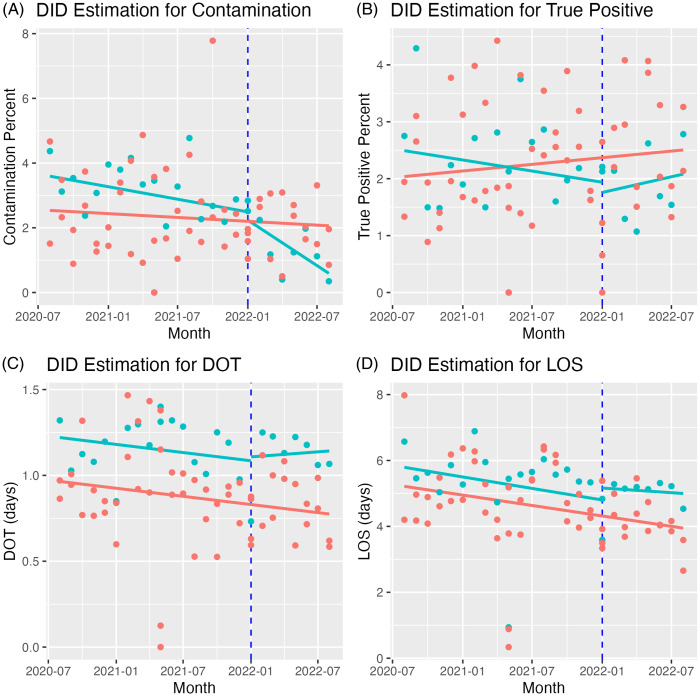
Difference-in-difference plots for study outcomes. This graph shows difference-in-difference plots for blood culture contamination (A), true positive blood culture (B), antibiotic days of therapy (days) for vancomycin and daptomycin (C), and length of stay (D). The green line represents the treatment group (center 1), while the red line represents the control group (centers 2 and 3). The blue dashed line represents the start of the second Plan-Do-Study-ACT cycle (PDSA2). Given the low initial specimen diversion device compliance in the treatment group during PDSA1, the pre-intervention and PDSA1 groups were combined.

The study included adult patients with a BCx collected on presentation to the emergency department or hospital admission. Only the first study encounter for each patient was included. The primary outcome was the BCC percentage. All hospitals had a standardized definition for BCC based on the National Healthcare Safety Network (NHSN) common commensals (Table A1).^4^ Secondary outcomes comprised true positive (TP) BCx percentage, hospital LOS (days), antimicrobial days of therapy (DOT) for vancomycin and daptomycin, and false-positive CLABSI. The latter was defined as any reported CLABSI to NHSN due to a single positive bottle for Coagulase-negative Staphylococci, enterococci, or *Candida* spp., based on Tompkins et al.’s definition.^2^

Outcomes were evaluated using single regression models: logistic (for BCC, TP BCx, and CLABSI), linear regression (for logarithmic values of LOS given non-normality), and zero-inflated negative binomial (for DOT given excess zeros and overdispersion using the pscl package^5^). We employed the difference-in-difference (DID) method in these models (treat*time) to estimate the effect of PDSA2 on the study outcomes by comparing outcome changes over time between the treatment and control groups. We combined the pre-intervention and PDSA1, given the low ISDD compliance in the treatment group. We then performed multiple regression models adjusting for the following variables (demographics, comorbidities, and BCx collection location). The DOT model included BCC as a covariate, while the log LOS model added DOT and BCC.

## Results

A total of 15,810 patients with BCx collected were included (5,441 pre-intervention, 5,734 PDSA1, and 4,635 PDSA2). Patient demographics were similar across the study periods.

The treatment group’s contamination percentage decreased from 3.83% (baseline) and 2.85% (PDSA1) to 1.32% in PDSA2 (DID –1.42, *P* = 0.011, Figure 1). The control group exhibited no notable change, remaining at 2.31%, 2.30%, and 2.17% for the corresponding periods. The difference in contamination percentages between the treatment and control groups remained statistically significant after adjusting for potential confounders (adjusted Odd Ratio [aOR] 0.40 [95% CI 0.28–0.56], Table 1).

**Table 1.** Multiple variable regression for study outcomes^*^

	BCC (Logistic)			TP (Logistic)			DOT (Count NB)			DOT (Zero-inflated NB)			Log Length of Stay (linear)		
*Predictors*	*OR*	*CI*	*p*	*OR*	*CI*	*p*	*IRR*	*CI*	*p*	*IRR*	*CI*	*p*	*Estimates*	*CI*	*p*
(Intercept)	0.03	0.02 – 0.04	**<0.001**	0.04	0.03 – 0.06	**<0.001**	3.79	3.25 – 4.41	**<0.001**	0.92	0.69 – 1.23	0.591	**1.48**	**1.41 – 1.55**	**<0.001**
Age (years)	1.01	1.01 – 1.02	**<0.001**	1.02	1.01 – 1.02	**<0.001**	1.00	0.99 – 1.00	**<0.001**	0.99	0.99 – 1.00	**<0.001**	**0.00**	**0.00 – 0.00**	**<0.001**
Gender [Male vs. Female]	1.17	1.02 – 1.34	**0.025**	1.22	1.05 – 1.40	**0.007**	1.10	1.04 – 1.17	**0.001**	0.83	0.75 – 0.91	**<0.001**	**0.03**	**0.06 0.01**	**0.008**
Race [Black vs. White]	1.16	0.99 – 1.35	0.058	1.19	1.01 – 1.40	**0.038**	0.91	0.85 – 0.97	**0.003**	0.90	0.80 – 1.01	0.066	**0.05**	**0.02 – 0.08**	**<0.001**
Race [Other]	0.77	0.54 – 1.05	0.113	1.35	1.01 – 1.77	**0.035**	0.89	0.78 – 1.01	0.061	0.94	0.76 – 1.17	0.589	**0.02**	**0.07 – 0.03**	**0.412**
SARS-COV2	1.33	1.11 – 1.58	**0.002**	0.79	0.64 – 0.97	**0.030**	0.90	0.82 – 1.00	**0.041**	2.21	1.92 – 2.54	**<0.001**	**0.23**	**0.20 – 0.27**	**<0.001**
SOT Listed vs. No	0.25	0.06 – 0.68	**0.020**	0.79	0.33 – 1.59	0.549	0.79	0.61 – 1.02	0.071	0.77	0.44 – 1.34	0.359	**0.02**	**0.13 – 0.09**	**0.752**
SOT [Yes]	0.51	0.35 – 0.73	**<0.001**	0.85	0.61 – 1.17	0.348	0.75	0.67 – 0.84	**<0.001**	0.67	0.50 – 0.90	**0.007**	**0.08**	**0.13 0.03**	**0.002**
Hemodialysis	1.38	0.22 – 4.71	0.665	3.28	0.95 – 8.70	**0.031**	0.53	0.28 – 0.98	**0.043**	0.37	0.05 – 2.85	0.341	**0.07**	**0.19 – 0.33**	**0.609**
HIV Positive	1.29	0.57 – 2.51	0.501	0.82	0.29 – 1.87	0.681	0.87	0.66 – 1.14	0.307	0.56	0.30 – 1.06	0.075	**0.09**	**0.23 – 0.04**	**0.183**
Neutropenia	0.94	0.52 – 1.56	0.817	2.89	1.94 – 4.17	**<0.001**	1.10	0.92 – 1.30	0.307	0.36	0.22 – 0.59	**<0.001**	**0.03**	**0.07 – 0.12**	**0.601**
Unit [ICU vs ED admit]	1.26	0.96 – 1.66	0.097	0.74	0.59 – 0.94	**0.013**	0.82	0.74 – 0.91	**<0.001**	1.52	1.19 – 1.93	**0.001**	**0.20**	**0.26 0.15**	**<0.001**
Unit [ED discharge]	0.80	0.59 – 1.09	0.160	0.73	0.56 – 0.94	**0.014**	0.34	0.29 – 0.41	**<0.001**	5.94	4.46 – 7.91	**<0.001**	**3.34**	**3.40 3.28**	**<0.001**
Unit [Ward]	0.61	0.47 – 0.79	**<0.001**	0.24	0.19 – 0.30	**<0.001**	0.90	0.81 – 0.99	**0.028**	2.50	2.01 – 3.12	**<0.001**	**0.50**	**0.55 0.44**	**<0.001**
BCC							1.04	0.92 – 1.16	0.554	0.75	0.60 – 0.94	**0.014**	**0.12**	**0.07 – 0.18**	**<0.001**
DOT													**0.09**	**0.09 – 0.10**	**<0.001**
treated	1.49	1.27 – 1.76	**<0.001**	1.03	0.86 – 1.22	0.768	0.79	0.74 – 0.85	**<0.001**	0.45	0.39 – 0.51	**<0.001**	0.08	0.05 – 0.11	**<0.001**
time	0.96	0.76 – 1.19	0.699	1.08	0.87 – 1.33	0.493	0.96	0.87 – 1.05	0.385	1.16	1.01 – 1.33	**0.040**	0.06	0.10 – 0.02	**0.001**
treated – time^#^	0.40	0.28 – 0.56	**<0.001**	0.66	0.48 – 0.90	**0.010**	1.04	0.92 – 1.18	0.559	0.82	0.66 – 1.03	0.086	0.03	0.02 – 0.08	0.261
Observations	16678			16678			16678			16678		16678		16678	
R^2^	0.015 (Tjur)			0.028 (Tjur)			0.159/ 0.158 (adjusted)			0.159/ 0.158 (adjusted)		0.654/0.654 (adjusted)		0.124/0.123	

BCC, Blood Culture Contamination; CI, 95% Confidence Interval; DOT, Days of Therapy; ED, Emergency Department; HIV, Human Immunodeficiency Virus; ICU, Intensive Care Unit; IRR, Incidence Rate Ratio; Log, Logarithmic; LOS, Length of Stay; NB, Negative Binomial; OR, Odd Ratio; SARS-COV2, Severe Acute Respiratory Syndrome Coronavirus 2; SOT, Solid Organ Transplantation; TP, True positive.

* The logistic regression model for central line-associated bloodstream infection (CLABSI) did not converge, and its results were not reported here.

# The treated*time variable represents the difference-in-difference (the effect of the intervention).

After the intervention, the percentage of true positive BCx decreased (OR 0.42, 95% CI 0.48–0.88). This reduction remained similar after adjusting for confounders (OR 0.66, 95% CI 0.48–0.90, Table 1). The intervention had no impact on DOT or LOS. There were three false-positive CLABSIs during the study (one in each period), and the CLABSI model did not converge due to low numbers.

## Discussion

We demonstrated that implementing ISDD resulted in a substantial reduction in BCC. This aligns with findings from other institutions employing similar techniques.^1–3^ However, our results showed no improvement in LOS, DOT, or the number of false-positive CLABSI.

Only a few studies utilizing ISDD have reported clinical outcomes. Callado et al.^6^ showed that hospital LOS, ICU LOS, and vancomycin DOT remained similar post-ISDD despite improved BCC. Conversely, Nielsen et al.^7^ demonstrated a 31.4% reduction in vancomycin DOT after ISDD implementation. Tompkins et al.^2^ reported lower false-positive CLABSI post-ISDD, though the standardized infection ratio had already been declining before the intervention. The difference in these studies’ results could be attributed to heterogeneity in designs, diversion techniques, and patient populations.

In our study, the percentage of TP decreased after the intervention. Conversely, another study demonstrated no change in TP after ISDD.^8^ This could be explained by the additional interventions at Center 1 restricting BCx to specific services (e.g., ICU) and selected indications.

An assumption of no realized cost reduction post-ISDD can be made, given the absence of improvement in measured outcomes. We hypothesize that the known association between LOS and BCC,^1^ which was also seen in our study (Table 1), could be attributed to unmeasured confounding effects (e.g., other comorbidities) rather than true consequences of BCC since a reduction in contamination did not lead to a decrease in LOS. Similarly, we did not see a reduction in DOT for vancomycin and daptomycin, as these antibiotics were likely given for indications other than BCC.

Several studies estimated cost savings using ISDD solely based on BCC reduction.^5^ Skoglund et al.^9^ provided a cost-benefit analysis projecting a cost saving of $272 per device, primarily by reducing LOS. Consequently, given the commercial ISDD cost, ISDD would not be cost-beneficial if the LOS reduction is not realized.

To our knowledge, this is the second study that assessed more than one ISDT. Arenas et al.^10^ found that both commercially available devices had lower contamination than the open technique. Similarly, our study showed lower BCC in the commercial device arm; however, it is possible that the difference in BCC was due to the rigorous training on aseptic techniques provided by the Steripath team rather than the diversion technique itself.

Our study has several limitations. The non-randomized design renders it unlikely to conclude a causal association. Although we have accounted for the role of external factors by providing a control group and adjusting for several confounders, there remain unmeasured confounding effects. We did not assess for echocardiogram utilization; however, they are rarely employed in patients with BCC. Moreover, we did not evaluate other relevant outcomes, such as the device’s ease of use or patient satisfaction. Finally, our study was restricted to Southeast Texas, limiting generalizability.

## Conclusion

We did not demonstrate a reduction in adverse clinical outcomes despite a marked decrease in BCC post-ISDD. This suggests that BCC’s association with these outcomes may reflect unmeasured confounding effects rather than a causative factor. Further research is required to elucidate the role of ISDD in patient care and hospital costs.

## Appendix

**Table A1.** Blood Culture Contamination Definitions

If blood culture isolate is an NHSN-defined “Common Commensal” (excluding *Staphylococcus lugdunensis, Corynebacterium jeikeium*, *Streptococcus anginosus*) and no other blood culture set collected within 24hr from blood culture set in question => “Single Set Contaminated.”
If a blood culture isolate (same species as in above) was isolated from a blood culture set collected within 72 hours from blood culture set in question => “True Positive”; otherwise, the blood culture growing Common Commensal should be regarded as “Contaminated” (Note: this includes instances where even a Gram-negative rod is present with the Common Commensal).

NHSN: National Healthcare Safety Network.
